# Supplementary material for: Identification of photoperiod-induced specific miRNAs in the adrenal glands of Sunite sheep (Ovis aries)
Source: Front Vet Sci. 2022 Jul 22;9:888207. doi: 10.3389/fvets.2022.888207 (PMC9354845; doi:10.3389/fvets.2022.888207)
Supplement: Supplementary file 1 [file Data_Sheet_1.ZIP › Supplementary file/miRDeep2 file.zip!/miRDeep2 file/Novel_Structure/SPLP42A3/Novel_647.pdf]

Provisional ID	:	NC_019473.2_27769
Score total	:	12.3
Score for star read(s)	:	3.9
Score for read counts	:	4.2
Score for mfe	:	2.5
Score for randfold	:	1.6
Score for cons. seed	:	
Total read count	:	20
Mature read count	:	19
Loop read count	:	0
Star read count	:	1

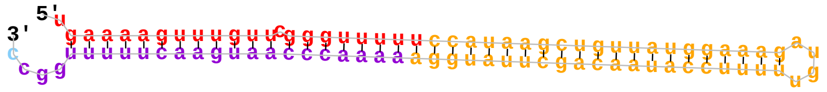

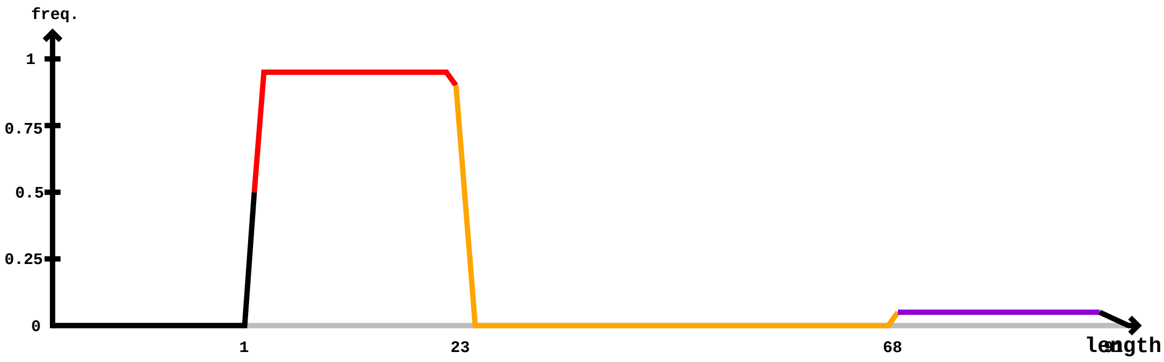

Mature   Star

```
5'- cccacagacacuggguuggugaaaaguuuguucggguuuuuccauaagcuguuauggaaagauguuuuuccauaacagcuuauggaaaaacccaaugaacuuuuuggccaa   -3'   obs
    cccacagacacuggguuggugaaaaguuuguucggguuuuuccauaagcuguuauggaaagauguuuuuccauaacagcuuauggaaaaacccaaugaacuuuuuggccaa         exp
    ((((.......))))(((((.(((((((((((.((((((((((((((((((((((((((((....)))))))))))))))))))))))))))))))))))))))..)))))   reads   mm   sample
    ...................ugaaaaguuuguucggguuuuu......................................................................   10      0    seq
    ....................gaaaaguuuguucggguuuu.......................................................................   1       0    seq
    ....................gaaaaguuuguucggguuuuu......................................................................   7       0    seq
    ....................gaaaaguuuguucggguuuuuc.....................................................................   1       0    seq
    ......................................................................................aaaacccaaugaacuuuuuggA...   1       1    seq
```
